# Probiotic Pre-treatment Reduces Gliclazide Permeation (*ex vivo*) in Healthy Rats but Increases It in Diabetic Rats to the Level Seen in Untreated Healthy Rats

**DOI:** 10.1111/j.1753-5174.2008.00006.x

**Published:** 2008-07

**Authors:** Hani Al-Salami, Grant Butt, Ian Tucker, Ranko Skrbic, Svetlana Golocorbin-Kon, Momir Mikov

**Affiliations:** * School of Pharmacy, University of OtagoDunedin, New Zealand; † Department of Physiology, University of OtagoDunedin, New Zealand; ‡ Department of Pharmacology, Medical Faculty, University of Banja Luka, Save Mrkalja Banja LukaBosnia and Herzegovina

**Keywords:** Probiotics, Gliclazide, Permeation, Transporters, Mrp2, Mrp3, Flux, Ussing Chambers, Diabetes, Rats

## Abstract

**Aim:**

To investigate the influence of probiotic pre-treatment on the permeation of the antidiabetic drug gliclazide in healthy and diabetic rats.

**Methods:**

Wistar rats (age 2–3 months, weight 350 ± 50 g) were randomly allocated into one of 4 groups (N = 16 each group): healthy control, healthy probiotic, diabetic control, and diabetic probiotic. Probiotics (75 mg/kg, equal quantities of *Lactobacillus acidophilus*, *Bifidobacterium lactis*, and *Lactobacillus rhamnosus*) were administered twice a day for three days to the appropriate groups after diabetes had been induced with alloxan i.v. 30 mg/kg. Rats were sacrificed, ileal tissues mounted in Ussing chambers and gliclazide (200 µg/mL) was administered for the measurement of the mucosal to serosal absorption Jss^(MtoS)^ and serosal to mucosal secretion Jss^(StoM)^ of gliclazide.

**Results:**

Treatment of healthy rats with probiotics reduced Jss^(MtoS)^ of gliclazide from 1.2 ± 0.3 to 0.3 ± 0.1 µg/min/cm^2^ (*P* < 0.01) and increased Jss^(StoM)^from 0.6 ± 0.1 to 1.4 ± 0.3 (*P* < 0.01) resulting in net secretion while, in diabetic tissues, treatment with probiotics increased both Jss^(MtoS)^ and Jss^(StoM)^fluxes of gliclazide to the comparable levels of healthy tissues resulting in net absorption.

**Discussion:**

In healthy rats, the reduction in Jss^(MtoS)^ after probiotics administration could be explained by the production of bacterial metabolites that upregulate the mucosal efflux drug transporters Mrp2 that control gliclazide transport. In diabetic rats, the restored fluxes of gliclazide after probiotic treatment, suggests the normalization of the functionality of the drug transporters resulting in a net absorption.

**Conclusion:**

Probiotics may alter gliclazide transport across rat ileal tissue studied ex vivo.

## Introduction

Probiotics are dietary supplements that contain live bacteria, which when consumed in adequate amounts, confer a health benefit on the host [1]. In order to improve the efficacy of probiotics, combinations of different bacterial strains can be used [2–4] with the mixture of *Lactobacillus* and *Bifidobacteria* being a common choice [3]. Probiotics are used in the treatment of a range of diseases such as infections, allergies, and inflammatory disorders [5–9]. However their use as a form of treatment for autoimmune diseases such as type 1 diabetes (T1D) is a current area of intense research and development [10].

Diabetic patients differ considerably in their response to antidiabetic drugs due to factors such as ethnicity, drug interactions and disease state [11,12], hence a thorough understanding of the pharmacokinetics and pharmacodynamics of antidiabetic drugs is essential to optimize individualized drug therapy. Drug optimization can also lead to fewer diabetic complications and a better quality of life [13]. Given the potential to use probiotics for the treatment of T1D, their integration with drug therapy should be explored further. Antidiabetic drugs are generally administered orally and vary considerably in their bioavailability [14–16]. This variation plays a significant role in their efficacy and safety [12,13]. Gliclazide is a second generation sulphonylurea used to treat non-insulin dependent diabetes mellitus (Type Π diabetes) [17]. Its primary mode of action is to induce insulin secretion by pancreatic β-cells [18,19] and as a result, it is ineffective, when administered alone, in the treatment of insulin dependent diabetes (Type I diabetes) [20].

**Figure 1:**
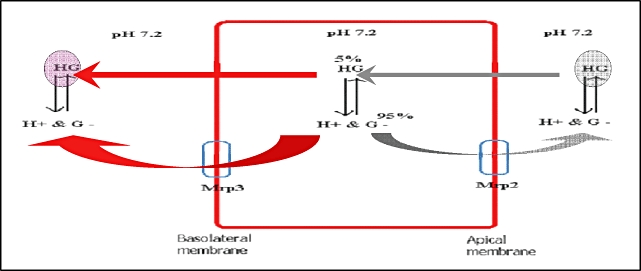
Gliclazide permeation through the ileal enterocytes through the action of the efflux transporters Mrp2 and Mrp3. G: gliclazide, G^-^: the negative ion of gliclazide molecule and H^+^: the proton produced from gliclazide ionization. Mrp: multi-resistance associated protein transporter. 5%: the percentage of the unionized gliclazide molecule inside the enterocytes (ileal mucosa). 95%: the percentage of the ionized gliclazide molecules inside the enterocytes.

The oral route is the most popular means for drug administration, since dosing is convenient and non-invasive [21]. However, the gastrointestinal mucosa represents a physical and biochemical barrier to the systemic availability of orally ingested, pharmacologically active molecules [22]. The function of the biochemical barrier depends largely upon the metabolism of drugs by intracellular enzymes and the operation of specific membrane transport systems. The efficacy of many drugs depends largely on their ability to cross cellular barriers to reach their target. However, the extent to which a drug reaches its targets within a tissue is limited not only by its ability to enter cells but by its tendency to depart due to the activity of efflux mechanisms in the plasma membrane. These efflux mechanisms such as the drug transporters Mdr1, Mrp2 and Mrp3, play a critical role in limiting the absorption and excretion of potentially toxic drugs and can effectively confer resistance to a diverse range of compounds [23]. Studying the influence of efflux drug transporters on drug permeability, in humans and animals, can be achieved by using Ussing chambers [24]. Transcellular absorption from lumen to blood requires drug uptake across the apical membrane, followed by transport across the cytosol, then exit across the basolateral membrane into blood [25]. Drugs that cross the apical membrane may be substrates for apical efflux transporters, which extrude compounds back into the lumen [26,27]. These apical efflux transporters are principally ABC transport proteins such as Mdr1, Mrp2, and Mrp3. These transporters pump out intracellular drugs or metabolites which they recognize [28]. Mdr1 and Mrp2 are located in the apical membrane of the intestinal epithelial cells and act as the first line of defence by limiting the absorption of potentially toxic compounds while Mrp3 are located on the basolateral membrane and remove their substrates from enterocytes to circulation (Figure 1). In a recent study, gliclazide absorption through the rat's ileum has been shown to be controlled by the efflux drug transporters Mrp2 and Mrp3 [29].

The changes in gut flora composition and gut motility have been reported in diabetes [30,31]. Accordingly, in this study we investigated the influence of treatment with probiotics on the permeation of the antidiabetic drug gliclazide in healthy and diabetic animals.

## Materials and Methods

### Materials

Gliclazide (99.92%) was purchased from Sigma Chemical Co, St Louis, MO, USA. ULTRA Water soluble transmission gel (hypoallergenic) was purchased from Medtel PTY. LTD. NSW, Australia. Freeze dried cultures of *Lactobacillus acidophilus*, *Bifidobacterium lactis*, and *Lactobacillus rhamnosus* were kindly provided by Professor John Tagg (Department of Microbiology, University of Otago) and Dr. Chris Chilcott (BLIS Technologies, Dunedin, New Zealand). All chemicals and solvents were of HPLC grade.

### Drugs Preparations

The gliclazide suspension 20 mg/mL was prepared by adding gliclazide powder to 10% Ultra water-soluble gel and mixing thoroughly at 37°C for 6 hours, and used within 48 hours of preparation.

### Animal Protocol and Ussing Chambers' Samples Preparations

The study was approved by the Otago University Animal Ethics Committee, New Zealand.

Male Wistar rats (age 3–5 months, weight 350 ± 50 g g) were randomly allocated into 2 groups, healthy (group 1) and diabetic (group 2), and were maintained in an experimental animal facility and given standard diet and water *ad libitum*. Temperature and light were controlled to mimic the natural habitat. Diabetes was induced by injecting alloxan (30 mg/kg) intravenously into the tail vein [32,33]. Rats were considered diabetics if blood glucose concentration was >20 mmol/L and serum insulin <0.04 µg/L, 2 to 3 days after alloxan injection [34–36]. Diabetic rats showed signs of polydipsia (abnormal thirst), polyuria (increased urination), weight loss (due to lean mass loss), and asthenia (weakness due to inability to utilise glucose) [33]. Healthy-probiotic (group 1) and diabetic-probiotic (group 2) were gavaged a freshly made mixture of *L. rhamnosus* GG, *L. acidophilus,* and *Bifidobacterium lactis*25 mg/kg each, twice daily for three days starting the next day after the onset of diabetes. Rats were sacrificed by CO_2_ then a midline longitudinal incision was made and the distal ileum (10 cm) was removed, flushed free of luminal contents with Ringer's (in mM: 140 Na^+^, 5.2 K^+^, 1.2 Ca_2_^+^, 1.2 Mg_2_^+^, 120 Cl^-^, 25 HCO_3_^-^, 2.4 HPO_4_^2−^ and 0.4 H2PO_4_^-^). The isolated ileum was mounted on a glass rod and the adherent tissues were carefully removed using a pair of blunt forceps. The Ringer's was then warmed to 37°C in a water bath and bubbled with carbogen (95% O2: 5% CO2) for 20 minutes to obtain a pH of 7.4 ± 0.05 prior to use. The excised section was opened along the mesenteric border, stripped off the underlying muscular layer, and mounted in Ussing chambers as flat sheets (exposed area 0.7 cm^2^). The pieces of the ileal mucosa (tissues) were placed between the two halves of the chambers and bathed by 10 mL of Ringer's on each side, and maintained at 37°C in water-jacketed reservoirs. Oxygenation and circulation was achieved by gas lift.

100 µL of gliclazide suspension (20 mg/mL) was added to the mucosal or the serosal side (final concentration = 200 µg/mL) of the Ussing chambers (at t = 0) and 20 µL samples were taken at −20, −19, 0, 10, 20, 30, 40, 60, 90, 120, and 180 minutes. Samples taken were immediately replaced by the same volume of Ringer's. Cumulative corrections were made for previously removed samples.

### HPLC and MS Analysis

The gliclazide concentrations in Ringer's was measured using high performance liquid chromatography (HPLC) based on the method of Park et al. and Rouini et al. [37,38]. Samples were mixed with acetonitrile in a 2:1 ratio, and after vortexing (for 20 seconds) and centrifuging (15,000 rpm for 15 minutes), the supernatant (20 µL) was injected into the HPLC system.

The column was a Luna 5 µm C18 (2) 100 × 2.00 mm from Phenomenex with a guard column (4 × 2.0 mm) also from Phenomenex. The detector was a Shimadzu, UV-V15 detector set at 229 nm. The mobile phase was acetonitrile 49% and water 51%, pH adjusted to 2.7 by orthophosphoric acid, at a flow rate of 0.4 mL/minute. The retention time for gliclazide was 2.9 minutes. A gliclazide standard curve was constructed using standard solutions of 0.5, 1, 2.5, 5, 10, 20, 40, 100, 200, 400 and 600 µg/mL. The within-day coefficient of variation ranged from 1.2% at 100 µg/mL to 2.9% at 0.5 µg/mL. The limit of detection was 0.4 µg/mL and the limit of quantitation was 0.8 µg/mL. The recovery rate of gliclazide from serum was 89 ± 4.1%.

Gliclazide samples were assayed once by MS, to assess gliclazide stability. MS was carried out on an LCQ Deca ion trap (Finnigan, Austin, TX, USA). Electrospray ionization in the negative ion mode was used. The instrumental parameters were: source spray voltage, 5.5 kV; capillary voltage, 44 V; heated capillary temperature, 150°C; sheath gas (nitrogen), 50 units. The MS/MS product ion spectra were produced by collision induced dissociation of the target molecular ions with optimized relative collision energy (CE) of 30 eV and isolated width (m/z) of 1. Excalibur version 1.2 (Finnigan) was used for data processing.

The steady-state flux (Jss) was based on the appearance of drug in the receiver (recipient) chamber under sink conditions:


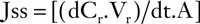


Where dCr/dt is the change in drug concentration in the receiver chamber at steady-state over time, Vr is the volume of receiver buffer (10 mL), A is the cross-sectional area of the exposed tissue (0.7 cm^2^). The flux ratio was calculated as the ratio of Jss of the mucosal to serosal over the serosal to mucosal, at equal drug concentrations.

Measurements of electrophysiological parameters of the mounted segments were made [39]. Transmural potential difference (PD) was short circuited by a dual voltage-clamp system (Biodesign, South Campus Electronics, University of Otago, Dunedin, NZ). The short circuit current (Isc) was corrected for fluid resistance. At the end of each experiment, tissues viability was tested by adding 30 mM glucose solution into the mucosal chamber and reporting the change in resistance (Ω.cm^2^) and Isc (µA/cm^2^). All data was from tissues which had a resistance and Isc >30 Ω.cm^2^ and 30 µA/cm^2^, respectively through out the experiment, and with >20 increase in Isc (µA/cm^2^) observed after the addition of glucose. Data were reported as mean ± standard deviation. Differences were considered significant if *P* < 0.05 using the nonparametric K-independent samples tests by SPSS (SPSS Inc. Version 13, USA) as well as the analysis of variance (anova) by Minitab (Minitab, Version 14; Minitab Inc, USA).

## Results

### The Effect of Probiotics on the Permeation of Gliclazide in Healthy and Diabetic Tissues

We have previously shown that in the ileum from healthy rats, the Jss^(MtoS)^of gliclazide was significantly greater than Jss^(StoM)^ flux resulting in the net absorption of gliclazide, which was most likely due to the activity of the efflux drug transporters Mrp2 and Mrp3. In contrast, in the ileum of diabetic rats, there was no net flux of gliclazide and the unidirectional fluxes were much smaller than those seen in healthy animals, suggesting a modification of the activity of Mrp2 and Mrp3 in type 1 diabetes.^40^ In the present study we demonstrate that the treatment with probiotics modified the gliclazide fluxes in both healthy control and diabetic animals. Accordingly, treatment with probiotics of healthy rats for three days reduced gliclazide permeation and increased its secretion through the ileum. This was a consequence of a six fold reduction (*P* < 0.01) of the Jss^(MtoS)^of gliclazide and a two fold stimulation of the Jss^(StoM)^of gliclazide (*P* < 0.01) compared to untreated animals. In contrast, in diabetic animals, treatment with probiotics stimulated the net absorption of gliclazide as a result of increasing Jss^(MtoS)^and reducing Jss^(StoM)^ (*P* < 0.01) to levels similar to that of healthy untreated animals (Table 1).

**Table 1:** Comparison between, the mucosal to serosal (M to S) and the serosal to mucosal (S to M) flux of gliclazide, at steady state (Jss), after treatment with probiotics, in tissues from healthy and diabetic rats. Tissues resistance through the course of the experiments, and the current change after glucose challenge, are shown. Data are mean ± SD

			Resistance (Ω.cm^2^) N = 32		Δ Short Circuit current (Isc) after glucose challenge (µA/cm^2^) N = 32	
Groups	Jss ^(M to S)^(µg/min/cm^2^) N = 16	Jss ^(S to M)^(µg/min/cm^2^) N = 16	M to S N = 16	S to M N = 16	M to S N = 16	S to M N = 16
Healthy (control)	1.18 ± 0.27	0.62 ± 0.14	41.03 ± 8.4	46.17 ± 10.6	30.36 ± 7.91†	33.14 ± 9.7†
Healthy-probiotic	0.26 ± 0.05*	1.38 ± 0.29*	55.03 ± 9.4	41.17 ± 8.6	35.9 ± 6.5†	29.21 ± 5.8†
Diabetic (control)	0.34 ± 0.09	0.35 ± 0.10	44.25 ± 9.97	45.39 ± 8.4	41.08 ± 6.23†	38.47 ± 8.14†
Diabetic-probiotic	1.26 ± 0.26*	0.66 ± 0.19*	50.25 ± 11.7	46.22 ± 10.6	37.4 ± 4.8†	40.36 ± 7.66†

* *P* < 0.01 gliclazide flux at steady state compared with the corresponding control.

† *P* < 0.01 Isc after glucose challenge compared with zero difference.

Collectively, our data show a change in gliclazide permeation through the ileum brought about by probiotic pre-treatment. This effect was different between healthy and diabetic rats.

## Discussion

In this study, we investigated the influence of treatment with probiotics on the ex vivo permeation of gliclazide, in healthy and diabetic rats, with the proposition that the chronic treatment with probiotics can change tissues permeability in healthy and diabetic animals. Gliclazide flux was used as an index of its permeation through the intestinal tissues and was measured using Ussing chambers.

We demonstrated in a previous study that gliclazide is a substrate of the ileal efflux drug transporters Mrp2 and Mrp3, and that these drug transporters are inhibited in the alloxan-induced diabetic rats with no net absorption of gliclazide [40]. In the present study, we have introduced the chronic treatment with probiotics, to investigate the influence of probiotics on gliclazide permeation in diabetic tissues. Our results show that chronic treatment of diabetic rats, with probiotics for three days, resulted in the net absorption of gliclazide through presumably the stimulation of Mrp2 and Mrp3, increasing both Jss^(MtoS)^ and Jss^(StoM)^ to the comparable levels of healthy tissues, while tissue resistance remained constant in all the groups throughout the course of the experiment.

Our results show that, in healthy tissues, treatment with probiotics markedly reduced Jss^(MtoS)^ (6 fold, *P* < 0.01). One potential explanation would be Mrp2 upregulation, which could reduce the absorption of gliclazide by extruding more gliclazide back into the mucosal solution. This upregulation of Mrp2 was supported when probiotic treatment increased the secretory unidirectional flux Jss^(StoM)^ of gliclazide in healthy tissues (2 fold, *P* < 0.01). Another possibility is the formation of a “thicker” layer of the adherent mucous as a result of the chronic treatment with probiotics [41], which increases the physical barrier protecting the enterocytes. This newly formed “bacterial” barrier may reduce gliclazide ability to reach the mucosal layer of the enterocytes and results in less gliclazide penetrating the enterocytes. Polypeptides originating from gut flora, such as *Lactobacillus*and *Bifidobacteria*have been shown to stimulate bacterial efflux drug transporters although a similar effect has not been shown in mammalian epithelial enterocytes [42–44]. Accordingly, in this study we postulate a similar effect on rat's ileal enterocytes, in particular the upregulation of the efflux drug transporters Mrp2 which we have shown, in a previous study [29] to control gliclazide ileal permeation. However, the study had significant limitations:

1. The expression of efflux transporters was not measured e.g., by immunohistochemistry or by blocking with a drug or antibody.
2. No controlled drug(s) was used which is known for being an Mrp substrates.
3. PK parameters were not measured.
4. Other cell cultures such as Caco-2 cells or Madin Derby Canine Kidney cells were not used to confirm results.

In conclusion, in the intestinal tissue of alloxan treated diabetic rats there is no net absorption of gliclazide across the ileal epithelium presumably due either to suppressed or malfunctional drug transporters. The results presented in this study demonstrate that the treatment with probiotics reversed the effect of alloxan-induced diabetes on ex vivo gliclazide transport, resulting in the net absorption of gliclazide across the tissue. In addition, when administered to healthy rats, probiotics treatment inhibited the absorptive and stimulated the secretory fluxes of gliclazide. In contrast, when applied to diabetic tissues, probiotics treatment induced an overall absorptive efflux through stimulating the net unidirectional flux, in the mucosal to serosal direction, and thus enhancing the net absorption of gliclazide across the tissue.

## References

[1] FAO/WHO (2002). Guidelines for the Evaluation of Probiotics in Food.

[2] Bezkorovainy A (2001). Probiotics: Determinants of survival and growth in the gut. Am J Clin Nutr.

[3] Karimi O, Pena AS (2003). Probiotics: Isolated bacteria strain or mixtures of different strains? Two different approaches in the use of probiotics as therapeutics. Drugs Today.

[4] Pena JA, Versalovic J (2003). Lactobacillus rhamnosus GG decreases TNF-alpha production in lipopolysaccharide-activated murine macrophages by a contact-independent mechanism. Cell Microbiol.

[5] Kruis W (2004). Review article: Antibiotics and probiotics in inflammatory bowel disease. Aliment Pharmacol Ther.

[6] Macfarlane GT, Furrie E, Macfarlane S (2004). Bacterial milieu and mucosal bacteria in ulcerative colitis. Novartis Found Symp.

[7] Marteau P, Lepage P, Mangin I, Suau A, Dore J, Pochart P, Marteau P, Lepage P, Mangin I, Suau A, Dore J, Pochart P, Seksik P (2004). Review article: Gut flora and inflammatory bowel disease. Aliment Pharmacol Ther.

[8] Bruzzese E, Canani RB, De Marco G, Guarino A, Bruzzese E, Canani R, De Marco G, Guarino A (2004). Microflora in inflammatory bowel diseases: A pediatric perspective. J Clin Gastroenterol.

[9] Dieleman LA (1997). Role of animal models for the pathogenesis and treatment of inflammatory bowel disease. Scand J Gastroenterol.

[10] Donohue DC (2006). Safety of probiotics. Asia Pac J Clin Nutr.

[11] Butler AE (2003). Beta-cell deficit and increased beta-cell apoptosis in humans with type 2 diabetes. Diabetes.

[12] Rendall M (2004). The role of sulphonylureas in the management of type2 diabetes mellitus. Drugs.

[13] Yaris F, Yaris E, Kadioglu M, Ulku C, Kesim M, Kalyoncu N, Yaris F, Yaris E, Kadioglu M, Ulku C, Kesim M, Kalyoncu N (2004). Normal pregnancy outcome following inadvertent exposure to rosiglitazone, gliclazide, and atorvastatin in a diabetic and hypertensive woman. Reprod Toxicol.

[14] Hansen JM, Christensen LK (1977). Drug interactions with oral sulphonylurea hypoglycaemic drugs. Drugs.

[15] Belcher G, Lambert C, Goh K, Edwards L, Valbuena M (2004). Cardiovascular effects of treatment of type 2 diabetes with pioglitazone, metformin and gliclazide. Int J Clin Pract.

[16] Bloomgarden ZT (2004). Glycemic treatment: Control of glycemia. Diabetes Care.

[17] Garcia-Bournissen F, Feig FS, Koren S (2003). Maternal-fetal transport of hypoglycaemic drugs. Clin Pharmacokinet.

[18] Campbell DB, Lavielle R, Nathan C (1991). The mode of action and clinical pharmacology of gliclazide: A review. Diabetes Res Clin Pract.

[19] Schernthaner G (2003). Gliclazide modified release: A critical review of pharmacodynamic, metabolic, and vasoprotective effects. Metab.

[20] Smith RJ (1990). Effects of the sulfonylureas on muscle glucose homeostasis. Am J Med.

[21] Werle M, Hoffer M (2006). Glutathione and thiolated chitosan inhibit multidrug resistance P-glycoprotein activity in excised small intestine. J Control Release.

[22] Li J, Hidalgo IJ (1996). Molecular modeling study of structural requirements for the oligopeptide transporter. J Drug Target.

[23] Chan LM, Lowes S, Hirst BH (2004). The ABCs of drug transport in intestine and liver: Efflux proteins limiting drug absorption and bioavailability. Eur J Pharm Sci.

[24] Lucas ML (2005). Amendments to the theory underlying Ussing chamber data of chloride ion secretion after bacterial enterotoxin exposure. J Theor Biol.

[25] Hunter J, Hirst BH (1997). Intestinal secretion of drugs. The role of P-glycoprotein and related drug efflux systems in limiting oral drug absorption. Adv Drug Del Rev.

[26] Evers R, Kool M, van Deemter L, Janssen H, Calafat J, Oomen L, Evers R, Kool M, van Deemter L, Janssen H, Calafat J, Oomen L, Paulusma C, Oude Elferink R, Baas F, Schinkel A, Borst P (1998). Drug export activity of the human canalicular multispecific organic anion transporter in polarized kidney MDCK cells expressing cMOAT (MRP2) cDNA. J Clin Invest.

[27] Fromm MF, Kauffmann H, Fritz P, Burk O, Kroemer H, Warzok R, Fromm M, Kauffmann H, Fritz P, Burk O, Kroemer H, Warzok R, Eichelbaum M, Siegmund W, Schrenk D (2000). The effect of rifampin treatment on intestinal expression of human MRP transporters. Am J Pathol.

[28] Dietrich C, Geier A, Oude R (2003). ABC of oral bioavailability: Transporters as Gatekeepers in the Gut. Gut.

[29] Al-Salami H, Butt G, Tucker I, Mikov M (2008). Influence of the semisynthetic bile acid (MKC) on the ileal permeation of gliclazide in healthy and diabetic rats. Pharmacol Rep.

[30] Iida M, Ikeda M, Kishimoto M, Tsujino T, Kaneto H, Matsuhisa M, Iida M, Ikeda M, Kishimoto M, Tsujino T, Kaneto H, Matsuhisa M, Kajimoto Y, Watarai T, Yamasaki Y, Hori M (2000). Evaluation of gut motility in type II diabetes by the radiopaque marker method. J Gastroenterol Hepatol.

[31] Drucker DJ (2007). The role of gut hormones in glucose homeostasis. J Clin Invest.

[32] Korec R (1980). Treatment of alloxan and streptozotocin diabetes in rats by intrafamiliar homo (allo) transplantation of neonatal pancreases. Endocrinol Exp.

[33] Carvalho IEN, Carvalho II, Ferreira LM (2003). Experimental model of induction of diabetes mellitus in rats. Acta Cirurgica Brasileira.

[34] Bachmann K, Pardoe J, White D (1996). Scaling basic toxicokinetic parameters from rat to man. Environ Health Perspect.

[35] Alam MJ, Rahman MA (1971). Changes in the saccharoid fraction in rats with alloxan-induced diabetes or injected with epinephrine. Clin Chem.

[36] Khavinson VK (2005). Effect of tetrapeptide on insulin biosynthesis in rats with alloxan-induced diabetes. Bull Exp Biol Med.

[37] Park J, Kim Y, Park K, Park P, Shin C (2003). Effect of rifampin on the pharmacokinetics and pharmacodynamics of gliclazide. Clin Pharmacol Ther.

[38] Rouini M, Mohajer A, Tahami M (2003). A simple and sensitive HPLC method for determination of gliclazide in human serum. J Chromatogr B Analyt Technol Biomed Life Sci.

[39] Boisset M (2000). Absorption of angiotensin II antagonists in Ussing chambers, Caco-2, perfused jejunum loop and in vivo: Importance of drug ionisation in the in vitro prediction of in vivo absorption. Eur J Pharm Sci.

[40] Al-Salami H, Butt G, Tucker I, Mikov M (2008). Influence of the semisynthetic bile acid MKC on the Ileal Permeation of Gliclazide *in vitro* in Healthy and Diabetic Rats treated with Probiotics. Methods Find Exp Clin Pharmacol.

[41] Bansil R, Stanley E, LaMont JT (1995). Mucin biophysics. Annu Rev Physiol.

[42] Nikaido H (1998). Multidrug efflux pump AcrAB of Salmonella typhimurium excretes only those beta-lactam antibiotics containing lipophilic side chains. J Bacteriol.

[43] Gunn JS (2000). Mechanisms of bacterial resistance and response to bile. Microbes Infect.

[44] Prouty AM, Van Velkinburgh JC, Gunn JS (2002). Salmonella enterica serovar typhimurium resistance to bile: Identification and characterization of the tolQRA cluster. J Bacteriol.

